# DOSIMETRY AND CANCER RISK ESTIMATIONS FOR DIFFERENT RADIATION PROTECTION SOLUTIONS AT DECOMMISSIONING A CONTAMINATED NUCLEAR POWER PLANT SITE

**DOI:** 10.1093/rpd/ncac192

**Published:** 2022-09-29

**Authors:** Martin Andersson, Keith Eckerman, Ünal Ören

**Affiliations:** Department of Radiation Physics, Sahlgrenska Cancer Center, University of Gothenburg, Gothenburg, Sweden; Medical Radiation Physics, ITM, Malmö, Lund University, SE-205 02 Malmö, Sweden; Center for Radiation Protection Knowledge, Oak Ridge National Laboratory, Oak Ridge, TN, USA; Barsebäck Kraft AB, Box 524, 246 25 Löddeköpinge, Sweden

## Abstract

Contaminated sediments originating from dredging activities in a nuclear power plant site were placed in a pond, which has to be taken into consideration during the future decommissioning process. The sediments have to be handled to free release the site. The radionuclides Co-60 and Cs-137 were identified and the activity concentrations (Bq/kg) were quantified in the range of 10–6000 and 5–50 Bq kg^−1^, respectively. The absorbed dose rate to individuals of various ages and sex present at the site of the dry pond area was estimated. The radiological impact in terms of lifetime attributable risk (LAR) and effective dose were calculated. For a 30-year-old male exposed during one year without any action regarding the sediments in the dried out pond, the LAR was predicted to be 0.0027, which recalculated to effective dose corresponds to 7.6 mSv year^−1^. The calculations show that countermeasures will be needed for the contaminated site.

## INTRODUCTION

There are many exposure situations where there is a need to determine internal and external doses for workers and the general public. In the nuclear industry, nuclear power plants such as Barsebäck and other Swedish sites will be decommissioned in the coming decades resulting in altered work environments and altered conditions for the general public. Furthermore, new knowledge about internal and external dosimetry are currently under development.

Dose limits, constraints and reference levels are set for planned, emergency and existing exposure situations, which are derived from environmental radionuclide concentrations. However, for information to the general public, there is a need for more detailed dose and risk estimations for sub-groups and even individuals. Just to tell that dose limits are kept has proved to be insufficient^(1, 2)^. In the context of patient exposure, it is well understood in that detailed dose and risk estimation is needed for subgroups of patients and even individuals^(3, 4)^. For diagnostic radiology and nuclear medicine, the cancer incidence per Sv for patients at 0–9 years of age is around twice as high as compared with patients at 30–39 years of age. For patients exposed in their 60s, the estimated lifetime risks are approximately half those for patients at 30–39 years^(3)^. Quantifying the excess radiation risk from the sediments gives the possibility to compare this risk with that of other risk to a normal population, e.g. from background radiation and from stable toxic elements such as arsenic, lead and cadmium.

### Effective dose and lifetime attributable risk

As an indicator to estimate the magnitude associated with radiation health risk, the effective dose (E) can be used. The effective dose is an important parameter used to justify the level of exposure of ionising radiation to occupationally exposed workers, in planned working conditions or to estimate the potential radiation induced risks for workers and the general public, e.g. in emergency situations. In the case of ingestion or inhalation, the internal dose to organs and thus the effective dose from a radionuclide depend on where the ionisation occurs, the energy imparted and the total number of disintegrations. To determine the total number of disintegrations a biokinetic model is created, where the radioactive substance is followed from intake until only an insignificant amount remains in the body. The committed effective dose, which is the effective dose integrated over 50 years, is commonly used in the context of internal exposure.

The effective dose is constructed to represent a detriment-adjusted risk, which accounts for morbidity and suffering of non-lethal cancers. The ICRP judges that cancer incidence should be weighted not only by lethality but also for pain, suffering and any adverse effects of cancer treatment. When defining the effective dose^(5)^, the total detriment adjusted nominal risk coefficient for cancer is 5.7% per Sv for the whole population and 4.1% per Sv for a population of workers.

An alternative to the use of effective dose is to use lifetime attributable risk (LAR). This approach has already been used in other situations, e.g. to describe the risk of cancer for patients in nuclear medicine^(6)^. The LAR approach is used by the United States National Research Council Committee to Assess Health Risks from Exposure to Low Levels of Ionizing Radiation (BEIR) VII^(7)^. The United States Environmental Protection Agency (EPA) has implemented a number of valuable extensions and modifications to the risk models given in BEIR VII^(8)^. For a given dose, LAR is the additional cumulated probability of having a specific cancer up to an age of 120 years. It relies on the use of a risk model derived from the epidemiological literature and is a classical risk indicator in the field of radiation protection. The EPA risk model predicts the risk of premature cancer death or incidence from radiation exposure for the US populations. The EPA risk estimations are generated from three different variables, age, sex and age at exposure, to predict the cancer risk. The estimations take into consideration that the secondary cancer risk is lower at higher ages and that there is a difference between risk for males and females. Also, ICRP recognises that there are significant differences in risk between males and females (particularly for breast) and with respect to age. But ICRP prefers a general system of protection, which is simple and sufficiently robust^(5, 9)^ and suitable as tool for radiation protection. The reader may wonder about the balance between the need for simplicity in application and the value of higher precision in risk management. As a first rough estimate of risk, the effective dose may be sufficient as an indicator of risk.

When better and more detailed dose estimates from external as well as internal exposure are available and information on future plans for the use of the land in question, there is a need for more detailed risk estimates.

Nuclear power plant sites contaminated with radionuclides such as Co-60 and Cs-137 pose a risk to human health through different exposure pathways such as direct external exposure or internal exposure due to inhalation and ingestion pathways. One of the goals of decommissioning of nuclear facilities is to reduce the residual radionuclide levels on site in order to meet free release criteria set by the competent authority. This study applied available site data for activity concentrations for a contaminated pond in a Swedish nuclear power plant for the effective dose, but also the sex- and age-specific LAR calculations. The contaminated sediments, originating from dredging activities, were placed in the pond during the second half of the 1990’s and first half of the 2000’s.

The aim of this project is to conduct realistic absorbed dose calculations from external exposure and estimates of the excess cancer risk for members of the public living on or close to a contaminated pond in a decommissioned power plant site. The external cancer risk will be based on the data primary data and models used in the EPA federal report No. 15^(10)^. To better estimate the cancer risk, the calculations in this work will not only determine the effective dose but also the sex- and age-specific LAR values.

## MATERIAL AND METHODS

### Radionuclide activity concentrations

The wet weight activity concentrations for gamma emitting radionuclides in sediment core samples (5-cm diameter and where the length of the sediment core samples were <45 cm) were previously collected from 10 individual locations in the pond, were measured and depth profiles were assessed. The sampling points were selected so that the sampling pattern would be representative for the whole pond. The sediment core samples were sliced into layers of 10 cm and the determination of activity concentrations (Bq kg^−1^ wet weight) was conducted by means of gamma spectrometry using a p-type high-purity germanium (HPGe) detector (EG&G Ortec) and using the Ortec GammaVision software for radionuclide identification and activity quantification. The gamma spectrometric system was calibrated using commercially available multi radionuclide standard sources. The depth profiles of the analysed radionuclides served as input parameters for assessing the radiological impact in terms of sex-specific (male and female) LAR of cancer incidence attributed to the exposure at various ages. The reference date for the activity quantification was 1st of January 2020.

### External dose calculations

**Figure 1:**
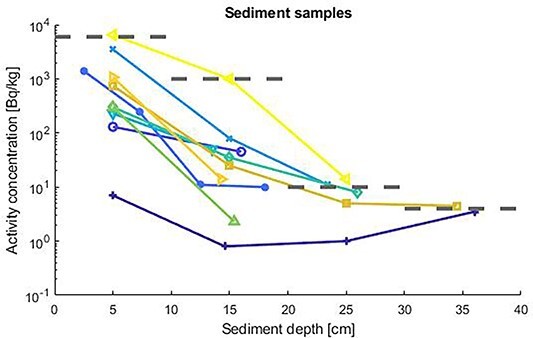
The wet weight activity concentrations in sediment samples for Co-60. The four grey dashed lines are the activity concentrations used in the calculations.

**Figure 2:**
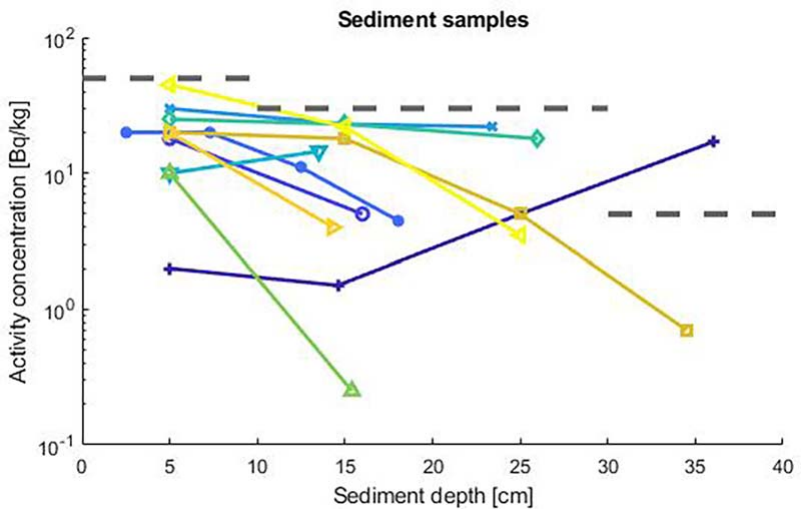
The wet weight activity concentrations in sediment samples for Cs-137. The three grey dashed lines are the activity concentrations used in the calculations.

FGR-15 is based on several different Monte Carlo simulations for three different exposure scenarios (air, soil and water). The Monte Carlo simulations are made for mono-energetic photons, enabling the possibility to perform organ calculations from 1252 radionuclides^(11)^. For soil calculations, the Monte Carlo simulations are performed at 5 different depth layers, allowing the possibility to calculate the external exposure from the surface or distributed down to an arbitrary depth and with different activity profiles. However, the FGR-15 report^(10)^ only includes data for uniform activity contamination in the soil present at the surface or for the depths of 1, 5, 15 cm and an infinite depth in the soil. The soil composition used in the calculations has a density of 1600 kg m^−3^ and a mass fraction composition of H 0.021, C 0.016, O 0.577, Al 0.050, Si 0.271, K 0.013, Ca 0.041 and Fe 0.011. As this project will use the primary data and models, it will be able to include the impact of removing contaminated sediments or adding non-contaminated soil to the contaminated areas. For long-time exposure from, e.g. Co-60 or Cs-137, there is also a possibility to change the exposure parameters during the integration time. For detailed information on the Monte Carlo simulations and the absorbed dose calculations, see FGR-15. The external calculations are performed on the sediment depth measurements shown in Figures 1 and 2, with a reference date of 01 January 2020. The effective dose and LAR values are calculated for three different hypothetical scenarios of a member of the public exposed for a year. First, calculations were performed for direct external exposure from the sediment. Thereafter, calculations were made where layers (between 1 and 15 cm) of the sediments were removed. Similar calculations were made for a situation when layers (between 1 and 15 cm) of ‘clean’ soil were added. The dose calculations also include the physical decay of the radionuclides^(11)^.

### Absorbed dose and effective dose calculation

The calculations assumes a uniform concentration $C(\tau )$ for an infinite or semi-infinite source region of a radionuclide at time τ. The equivalent dose ${H}_T({T}_{Age},{T}_{Exp})$ in tissue $T$ of an individual of age ${T}_{Age}$ during an exposure of duration ${T}_{Exp}$which can be expressed as

(1) \begin{equation*} {H}_T\left({T}_{Age},{T}_{Exp}\right)={\int}_0^{T_{Exp}}C\left(\tau \right)\ast \dot{h_T}\left({T}_{Age}+\tau \right)\ d\tau \end{equation*}

where $\dot{h_T}(\tau )$ is the time-dependent dose rate coefficient for external exposure. The coefficient $\dot{h_T}$ represents the dose rate to tissue $T$ of the body of an individual of initial age ${T}_{Age}$ per unit time-integrated exposure expressed in terms of the time-integrated concentration of the radionuclide. This coefficient varies with time due to anatomical changes as the receptor grows from a newborn to an adult^(10)^.

The effective dose ($E$), which is the sum of sex average radiation weighted equivalent dose from radiosensitive organs is calculated by

(2) \begin{align*} &E = {\sum}_T{w}_T \nonumber \\ &\kern-3pt\times\frac{H_{T, Male}\left({T}_{Age},{T}_{Exp}\right)+{H}_{T, Female}\left({T}_{Age},{T}_{Exp}\right)}{2}\ \left[ Sv\right] \end{align*}

where ${w}_T$ is the tissue weighting factor representing the relative organs and tissues detrimental effects. ${H}_{T, Male}({T}_{Age},{T}_{Exp})$ and ${H}_{T, Female}({T}_{Age},{T}_{Exp})$ are the mean equivalent dose of target region $T$ of the reference male and female person, respectively^(4)^.

### LAR calculations

The $LAR$ cancer risk predictions for each of the 15 specific cancer sites are presented in Eq. 3. The calculations are performed in three steps. The first step is to calculate different cancer types, age specific excess rate of cancer diagnosis, $M(D,e,a)$. The $M(D,e,a)$ is a function of three variables, the time dependent absorbed dose ($D$) of the specific organ, the age ($e$) at the exposure and the attained age ($a$) of cancer diagnosis. The next step is to multiply the excess cancer rates by the probability of being alive at age $a$, $S(a)$ each year after the exposure, normalized by the probability of being alive at exposure. Finally, the $LAR$ is obtained by integrating these adjusted excess cancer rates over attained age starting post a latent period of 5 years (two for leukemia) after the exposure.

(3) \begin{equation*} LAR{\left(D,e\right)}_{Sex}={\int}_{e+L}^{110}M\left(D,e,a\right)\bullet \frac{S(a)}{S\left(e+L\right)} da \end{equation*}

where $D$ is the absorbed dose, $e$ is the age (year) at exposure, $L$ is the latency period (year) after exposure for which stochastic effects occurs, $a$ is the attained age (year), $S(a)$ and $S(e)$ are the survival rate at ages $a$ and $e$, respectively^(7)^.

## RESULTS AND DISCUSSION

### Activity concentrations

The radionuclides Co-60 and Cs-137 were identified and the activity concentrations versus sediment depth are shown in Figures 1 and 2. The samples contained up to 6 kBq/kg Co-60 and 0.05 kBq/kg Cs-137. The results for the different sediment depths showed that the activity levels were concentrated at the uppermost 10 cm levels. The dose calculations were based on conservative estimates of the activity concentrations, especially for deep sediments. Generally, for the most of the samples, it is observed that the activity concentration profiles peaks within the first 10 cm and thereafter decreases with sediment depth due to processes such as radionuclide migration, although the decreasing rate is more pronounced for Co-60 and in one of the samples an increase is noted for both Co-60 and Cs-137.

### External dose calculations

**Figure 3:**
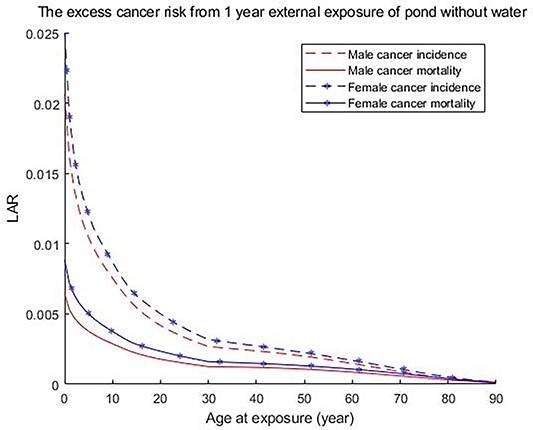
Age at exposure and sex-dependent cancer morbidity and mortality risks from 1-year fulltime external exposure from the pond without water and any actions regarding the sediments.

The current scenario focuses on the external exposure pathway, where the individual is assumed to be subject to direct exposure from radionuclides in the sediments and where the individual is assumed to spend the whole year on the contaminated site and where the outdoor fraction is 100%. Three different hypothetical scenarios with respect to different management options for the contaminated sediment were calculated from the sediment depth profiles in Figures 1 and 2. Effective doses for an exposed individual during 1 year without any adjustment of the sediment and when the pond is emptied of water are shown in Table 1 for 6 different age groups and for Co-60 and Cs-137. It is noted that the effective dose is dominated by Co-60. The effective dose calculations are conservative since it is assumed that the particular individual spends the whole year on the contaminated pond. The used upstanding posture simulated data^(10)^ of for the new-born and 1-year-old given in Table 1 is not the best representation for that age group but can used as a rough indicator of dose levels. Figure 3 gives additional information on the age-specific radiation risk. It is important to note that the risk for families with children or those who are expected children is significantly higher than for families with only adults.

**Table 1:** The effective dose for exposure of 1-year fulltime exposure from the pond without water and no modifications made on the dried out sediments

Nuclide			Effective dose [mSv]			
	New-born	1 year	5 years	10 years	15 years	Adult
Co-60	9.9	9.2	8.7	8.3	7.8	7.6
Cs-137	0.000013	0.000012	0.000010	0.000010	0.0000086	0.0000084

The corresponding LAR values are shown in Figure 3. As an example, for a 30-year-old male exposed during 1 year without any adjustment of the soil and when the pond is emptied of water the excess cancer incidence risk was predicted to be 0.0027. This corresponds to an annual effective dose of 7.6 mSv. The corresponding values for a 30-year-old female were 0.0032.

**Figure 4:**
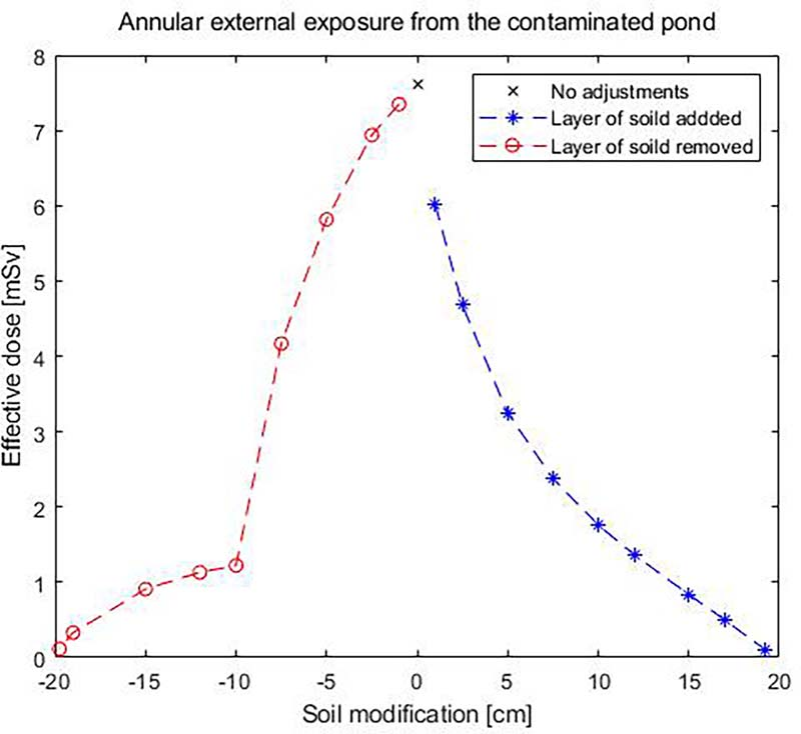
The effective dose for exposure of 1-year fulltime exposure from the dried out pond when different amount of ‘clean’ soil is added and sediments removed, respectively.

Thereafter, calculations were made where layers of the sediments were removed or where layers of soil were added on the sediments. The effect of the effective dose by adding soil or removing layer of sediments are shown in Figure 4.

In Figure 4, the effect of varying the amounts of removed sediment versus adding soil on the contaminated sediment is illustrated. To be able to free release the area, the Swedish Radiation Safety Authority has set an annular dose limit of 0.1 mSv to members of the public. It is shown that this dose limit is reached by either removing 19 cm of sediment or adding 20 cm of clean soil on the sediment. In practise, the solution of adding soil will be problematic since the cover can be redistributed by future agriculture or gardening.

**Figure 5:**
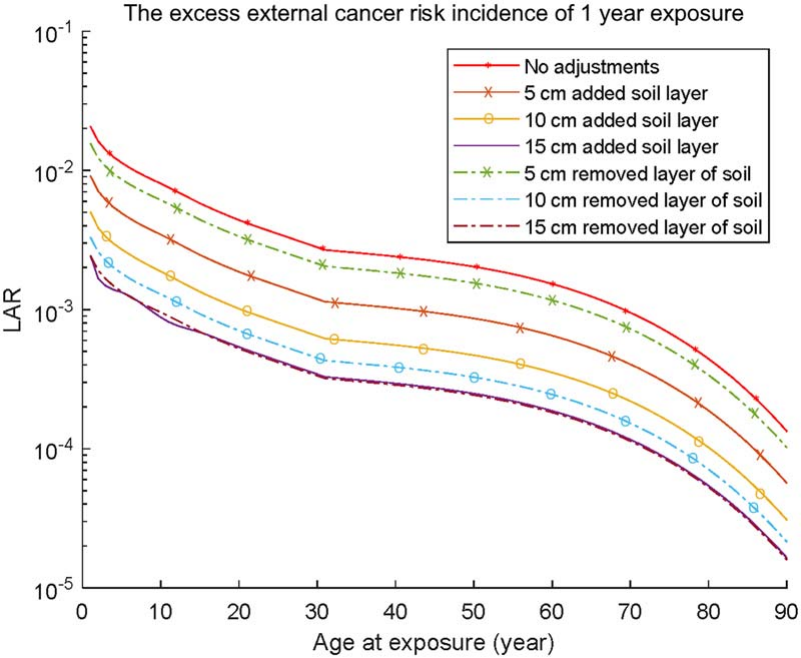
The increased cancer risk for exposure at various ages (0–90 years) of 1-year fulltime external exposure from the pond without water when different amount of soil is added or sediment is removed, respectively.

The same calculations were performed for LAR and shown in Figure 5, e.g. by adding 5 cm of soil on the surface will accomplish a dose reduction of 57% or by removing 5 cm of sediment will accomplish a dose reduction of 23%. As nuclear power plants are decommissioned, there is a need to perform cost–benefit analyses regarding radiological risks and different management solutions for areas with residual activity.

To achieve the regulatory dose limits for areas, different countermeasures or combination of countermeasures could be undertaken. These include removal of the sediment or adding clean soil on the contaminated sediment. Based on the LAR calculations, different actions, such as those mentioned above, could be implemented in order to reduce the contamination levels in the pond and to minimize the radiation doses. Future work in this area should also involve considering different exposure pathways such as inhalation of resuspended dust and ingestion of contaminated sediment particles.

Also, derived concentration guideline levels corresponding to the contamination levels that result in an effective dose equivalent to the site release criteria should be determined. In the Swedish regulations, it is stated that for unrestricted reuse scenario, the effective dose received by members of the public being exposed to residual radioactivity shall not exceed 0.1 mSv y^−1(^^12^^)^ where the relevant exposure pathways have to be identified and considered. These exposure pathways constitute external exposure by soil contamination and internal exposure due to inhalation and ingestion of contaminated dust. Other pathways may include for example ingestion of drinking water and consumption of fruits, vegetables, meat, fish, milk and other relevant foodstuff related to the specific site.

## CONCLUSION

Since the shutdown of several nuclear power plants in Sweden, preparatory activities for decommissioning are carried out where an important part is the final release of the sites. This study demonstrated an alternative risk assessment method to that of effective dose and the results from different countermeasure options where the residual activity levels are reduced.

The results indicate that for external exposure point of view, a level of 0.1-mSv annual effective dose could be achieved by either removing ~20 cm of contaminated soil or by adding the same amount of clean soil. The LAR methodology described in this work will facilitate a basis for the future management of the contaminated pond.

## FUNDING

This project was funded by Swedish Radiation Safety Authority (grant number: SSM2020-642).
